# Is telephone follow-up really effective in early diagnosis of inflammatory complications after tooth extraction?

**DOI:** 10.4317/medoral.22465

**Published:** 2018-11-21

**Authors:** Roberto Pippi, Alessandra Pietrantoni, Romeo Patini, Marcello Santoro

**Affiliations:** 1 MD, DDS, Associate Professor, Department of Odontostomatological and Maxillo Facial Sciences, “Sapienza” University of Rome; 2 DDS, Department of Odontostomatological and Maxillo Facial Sciences, “Sapienza” University of Rome; 3 DDS, PhD, Department of Surgical Sciences for Head and Neck Diseases - Catholic University of Sacred Heart - Rome, Italy; 4 DDS, PhD, Department of Odontostomatological and Maxillo Facial Sciences, “Sapienza” University of Rome

## Abstract

**Background:**

To establish whether telephone follow-up is really able to intercept post-extraction complications and to evaluate the degree of patient satisfaction with this kind of post-surgical monitoring.

**Material and Methods:**

Six hundred and thirty-eight patients were enrolled and randomly assigned to a test or control group. Test group patients were monitored by telephone follow-up 24 and 72 hours after surgery to investigate the presence of local symptoms that are frequently associated with surgical wound infection and inflammation. Both test and control group patients were examined 7 days at suture removal. Patients with systemic diseases, those in which intra-operative accidents occurred during surgery and those for whom extraction suture was not required, were excluded.

**Results:**

At least one complication among alveolar osteitis, alveolar inflammation, alveolar infection and dehiscence involved 15.70% of the patients in the test group and 30.70% of the patients in the control group and telephone follow-up proved to be useful in early identification of anomalies in the post-extraction wound healing process. Comparable results were recorded in all extraction subgroups divided according to the type (surgical and non-surgical) and the number (single and multiple) of extractions performed in the same session. Telephone follow-up showed an 8.60 ± 1.17 (0 to 10 score scale) average acceptance. All cases of alveolar osteitis and infection occurred in patients who underwent antibiotic prophylaxis.

**Conclusions:**

Telephone follow-up seems to allow early detection of any possible wound healing complications, it is widely accepted by patients and it could therefore be considered a valid method for wound healing monitoring after tooth extractions, due to its effectiveness, feasibility and low costs.

** Key words:**Oral surgery, complications, telephone follow-up.

## Introduction

Post-operative control examinations following tooth extractions are aimed to intercept and prevent post-operative complications (1). However, since complications occur in a small percentage of cases (5-20%) (2) and mostly in patients with specific risk factors which should be pre-operatively identified (3), clinical monitoring has been considered by many authors as a waste of time with questionable benefits for patients (4,5).

Alveolar osteitis and infections are the most serious complications associated with tooth extractions. Although infrequent if untreated or incorrectly or belatedly treated, they may lead to severe (2,3,6,7) and life-threatening (2-4,6,7) complications with airway, mediastinal and vertebral involvement, sepsis, and blindness as a result of cavernous sinus thrombosis, and to other conditions that can be prevented with early and effective treatment (4), thus making post-operative monitoring necessary.

Considering the low rate of post-operative complications and their easy detectability as well as the high costs involved in clinical follow-up of patients who have had teeth extracted, telephone follow-up (6) has been considered a viable alternative for patients and national health facilities worldwide (4,8,9).

The primary aim of the present study was therefore to verify if telephone follow-up allows to intercept post-extraction complications and to evaluate the degree of patient satisfaction with this kind of post-surgical monitoring. The second aim was to evaluate if any correlation exists between antibiotic prophylaxis and surgical site infections (2-4,10).

## Material and Methods

In the present study, patients undergoing at least one tooth extraction over a 18-month period of time in the Oral Surgery Unit of the Head-Neck Integrated Care Department of Umberto I hospital-polyclinic University of Rome were included. Patients were randomly assigned to test and control groups. Test group patients were monitored by telephone follow-up, whereas the control group patients were not monitored at all during the first week after surgery. Patients of both groups were examined at the time of suture removal 7 days following surgery considering that the outpatient clinic where the research took place was open on a Monday to Friday basis.

Since at the time the present study was being set up only one retrospective study was present in the dental scientific literature reported data on the different incidence of post-operative infection in patients followed by telephone or clinical follow-up, with an incidence of 9.68% in 155 cases and 17.22% in 209 cases, respectively, in the present study, a 10% incidence of events in the test group and 18% in the control group were assumed. A total of 638 patients, 319 per group, were therefore set to be enrolled since this sample showed an 80% power and a 5% significance level.

The following patients were excluded from the study: patients with systemic diseases, patients enrolled in other studies, patients in which intra-operative accidents occurred during surgery, patients for whom extraction suture was not required.

At the time of surgical planning, patients were asked to participate in the study and protocols and goals were explained to them. It was also specified that they were free to participate or not and all participants signed the consent form for the study participation and to permit the use of their personal data for the study.

All extractions were performed under local anesthesia according to standard protocol by experienced surgeons and post-graduate students in oral surgery. Patient assignment to the test or control groups, with the respective identification codes, was performed randomly at the end of surgery by an external operator. All patients received the same post-operative instructions. Test group patients were contacted by phone 24 and 72 hours after surgery and the interviewer gave them a questionnaire that was included in a clinical chart with patient personal data, the phone number they preferred to be contacted at, how their tooth was extracted, pre-operative symptoms and whether and how antibiotic prophylaxis was performed. The questionnaire included both multiple choice open and closed questions and it was designed to investigate the presence of all local symptoms that are frequently associated with both alveolar and deep-tissue infections such as pain, bleeding, dysphagia, trismus and systemic symptoms related to infection like fever. Pain and swelling were recorded using a 0-10 numerical rating scale while trismus and dysphagia were registered with a 3-grade verbal scale (slight-moderate-severe). Patients were asked for their own opinion on wound condition/healing and if they contacted their family doctor for any related problems. It was expected that in cases of suspected infection patients were invited to come in for examination before suture removal. At the time of suture removal patients were asked if they were willing to accept telephone follow-up, if they would have preferred returning for examination rather than be contacted by phone and if they wanted to report any other wound-related issues.

Patients belonging to the control group were only seen at the time of suture removal and a questionnaire was filled out to report any possible wound problems or any other information during the week after surgery which could indicate the presence of post-operative complications or problems prior to examination.

Clinical evaluation of post-extraction socket healing was based on the following conditions and criteria:

-Alveolar osteitis: persistent or increasing post-operative pain not relievable with analgesics, presence of an empty socket or a partially or totally disintegrated blood clot, halitosis.

-Acutely inflamed socket: a painful socket with inflamed tissue but without exudate or fever.

-Acutely infected socket: a painful socket with suppuration, erythema and edema, with or without systemic fever.

-Normal healing socket: presence of normal granulation tissue with or without pain.

Descriptive statistics were performed for all study variables. Independent sample t-test and chi-square Fisher’s test were performed to assess relationships among continuous data and categorical measures, respectively. Bivariate logistic regression analysis was performed to assess the relationship between the study groups (test and control) and post-operative complications. For all analyses, *p* ≤ 0.05 was considered significant.

The study was approved by the local Ethical Committee - Protocol Number 2755.

## Results

For a better descriptive statistical analysis, the two groups (test and control) were divided into 4 subgroups according to the type (surgical/non-surgical) and the number (single/multiple) of extractions performed in the same session.

Extractions were considered surgical when an access flap was required for them to be performed. In the surgical extraction group, surgeries in which at least one of the extractions was surgical were included whereas in the non-surgical group none of the extractions performed were surgical. The single extraction group included patients who had only one extraction, whereas the multiple extraction group included patients who had more than one extraction.

Descriptive analysis of study samples and their subgroups are summarized in  Table 1,  Table 1 continue. The overall average age of the study sample was 36.09 ± 16.62 (35.04 ± 14.90 for the test group and 37.15 ± 18.14 for the control group, *p* = 0.15, CI = 95%).

**Table 1:**
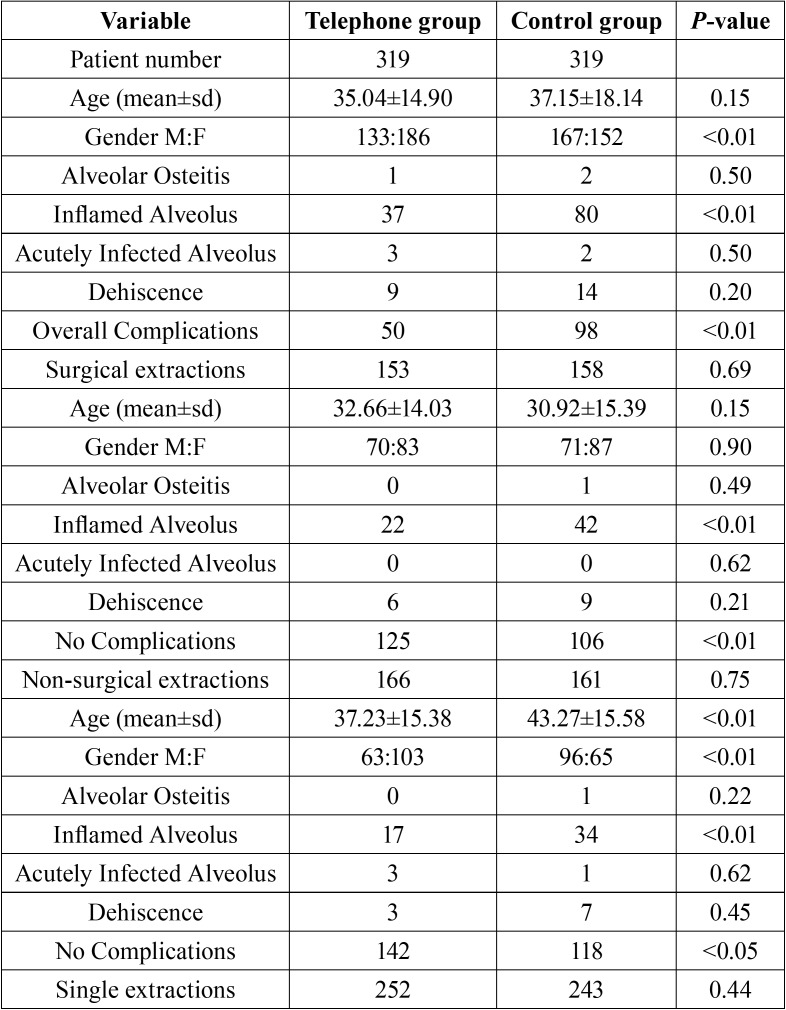
Overall study sample features and post-operative complications in telephone follow-up group and control group.

**Table 1 continue:**
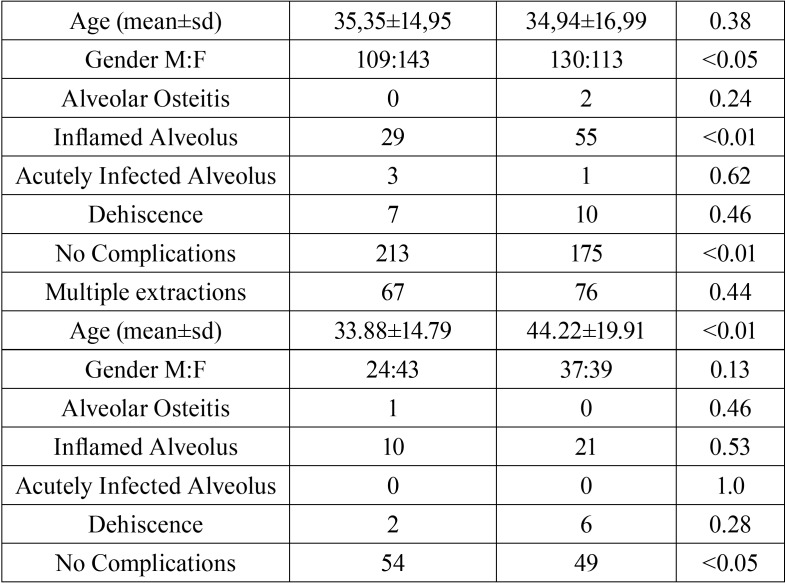
Overall study sample features and post-operative complications in telephone follow-up group and control group.

In the control group 158 (49.5%) patients and in the test group 153 (47.9%) patients underwent surgical tooth extractions (*p* = 0.69, CI = 95%). Seventy-six (23.8%) patients in the control group and 67 (21%) patients in the test group underwent multiple extractions during the same session (*p* = 0.44, CI = 95%).

As for the main aim of this study, at least one complication, among alveolar osteitis, alveolar inflammation, alveolar infection and dehiscence, involved 15.70% of the patients in the test group and 30.70% of the patients in the control group and the telephone follow-up proved to be useful for early identification of anomalies in the post-extraction wound healing process (*p* <0.01, OR 0.4365, 0.2982-0.6390, 95% CI). Comparable results were recorded in all subgroups of extractions ( Table 2).

**Table 2:**
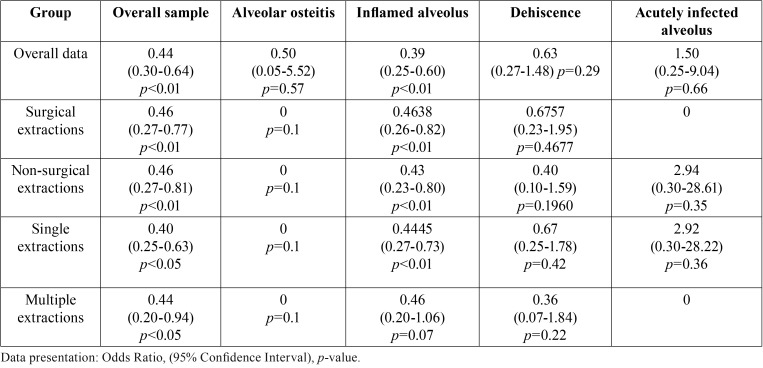
Bivariate associations between telephone follow-up and post-operative complications (significant p-values are highlighted in red).

Alveolar osteitis involved 0.31% of patients in the test group and 0.62% in the control group (*p* = 0.50, CI = 95%), without a positive association with telephone follow-up (*p* = 0.5705, CI = 95%). This result also showed no statistically significant variations between the two groups with regard to surgical extractions, non-surgical extractions, multiple extractions and single extractions ( Table 2).

Alveolar infection involved 0.94% of patients in the test group and 0.62% in the control group (*p* = 0.50, CI = 95%), without a positive association with the telephone follow-up ( Table 2, *p* = 0.6581, CI = 95%). Analogous association was found in each extraction subgroup.

Alveolar inflammation occurred in 11.60% of patients in the test group and 25.07% in the control group (*p* <0.01, CI = 95%) and telephone follow-up played an important role in early identification of post-extraction wound inflammatory processes (*p* <0.01, OR 0.3898, 0.2545-0.5971, CI = 95%), as well as in all subgroups of extractions, excluding multiple extractions ( Table 2).

A wound dehiscence at the extraction site involved 2.82% of patients in the test group and 4.40% in the control group (*p* = 0.2067, CI = 95%) without any positive association with telephone follow-up ( Table 2) (*p* = 0.6303, CI = 95%), as well as within all extraction subgroups.

Eighty-four % and thirty two percent of patients in the test group and 69.27% in the control group generally displayed normal post-extraction healing without any complications (*p* = 0.056, CI = 95%). As for the extraction subgroups, healing was normal after surgical extractions in 81.70% of patients in the test group and in 67.10% of patients in the control group (*p* <0.01, CI = 95%), in 85.54% and 73.29% respectively, after non-surgical extractions (*p* <0.01, CI = 95%), in 84.52% and 72.01%, respectively, after single extractions (*p* <0.05, CI = 95%) and, finally, in 80.60% and 64.47%, respectively, after multiple extractions (*p* <0.01, CI = 95%).

In relation to the telephone questionnaire ( Table 3,  Table 3 continue), 24 hours after surgery 26.96% of the test group patients perceived no pain, whereas 39.18% perceived a 7-8 subjective pain intensity and only 1.88% reported maximum intensity pain (10). Therefore, 60.81% of patients needed to use pain-relievers and/or anti-inflammatory drugs. After 72 hours, 48.27% of patients reported no painful symptoms and 38.56% reported a pain intensity ranging from 3-6. Only 0.31% of patients still reported maximum intensity pain. The use of pain-relievers or anti-inflammatory drugs was reported by 36.36% of patients. 5-7 days after surgery, 81.81% of patients reported no pain and 9.09% reported a 2-3 pain intensity. No patients reported intensity ranging from 7-9 and 1 patient (0.31%) still reported a 10-score pain intensity. About 9% of patients reported they needed to continue using pain-relievers and/or anti-inflammatory drugs.

**Table 3:**
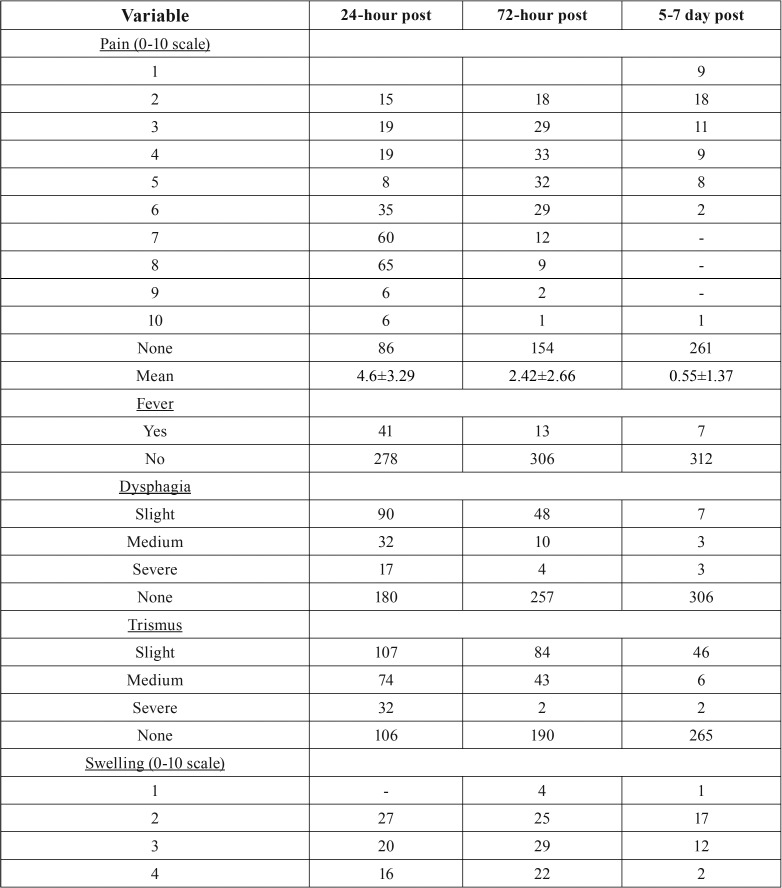
Overall features of the telephone follow-up group questionnaire.

**Table 3 continue:**
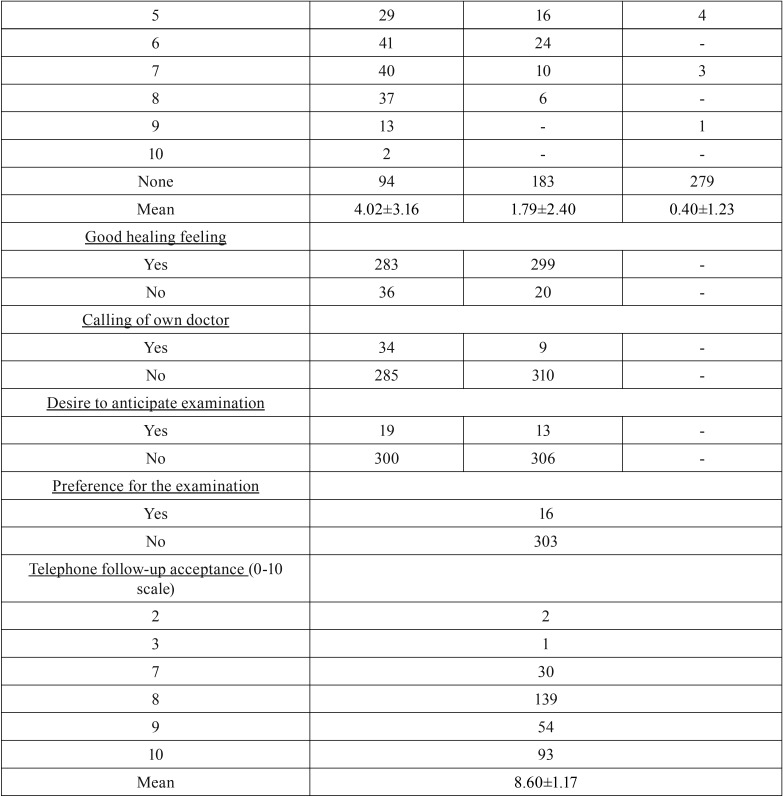
Overall features of the telephone follow-up group questionnaire.

Only 5.33% and 1.25% of patients reported severe difficulty and intense pain during swallowing after 24 and 72 hours, respectively. Less than 1% of patients continued to perceive these symptoms at 5-7 days after surgery while 56.42%, 80.56% and 96% of patients reported no dysphagia at 24, 72 hours and 5-7 days after extraction, respectively.

A moderate to severe difficulty in mouth opening (trismus) was reported by 33.23% and 4.40% of patients at 24 and 72 hours, respectively, and in 2.51% of patients it extended to 5-7 days. On the contrary, trismus was not reported by 33.22%, 59.56%, and 83.10% of patients at 24, 72 hours and 5-7 days after extraction, respectively.

Objective swelling scores were 6-7 at 24 hours in 25.39% of patients, and 10 in 0.63% of patients, whereas 29.47% of them had no swelling. At 72 hours and 5-7 days after extraction, 57.36% and 87.46% of patients, respectively, reported no swelling and only 9.09% reported subjective swelling between 2 and 3.

Fever was reported by 12.85% and 4.07% of patients 24 and 72 hours after surgery, respectively, persisting to 5-7 days in 2.19%.

“Normal” healing was reported by 88.71% and 93.73% of patients at 24 and 72 hours after surgery, respectively, while only 10.65% of patients reported having had the need to consult their family doctor after 24 hours and 2.82% did so after 72 hours. However, 72 hours after surgery, approximately 95% of patients did not require to move up their check-up appointment nor did they report any preference for clinical monitoring rather than telephone monitoring. No patient returned with late infection. Finally, telephone follow-up showed an 8.60 ± 1.17 average acceptance.

As for the second aim of this investigation, 46.23% of the entire study sample underwent antibiotic prophylaxis. In this group of patients, inflammatory complications occurred in 34.23% of cases. In patients who did not undergo antibiotic prophylaxis (53.77%), this type of complication occurred in 13.40% of cases. In particular, within the subgroup of surgical extractions, the frequency of antibiotic prophylaxis was higher (52.09%) than within the subgroup of non-surgical extractions (40.67%) and at least 1 complication involved 35.6% of patients who underwent antibiotic prophylaxis and 14.7% of those who did not. Finally, all cases of alveolar osteitis and infection occurred in patients who underwent antibiotic prophylaxis.

## Discussion

The present study seems to show that telephone follow-up allows early identification of some post-operative complications. The most effectively identified complication was alveolar inflammation in both surgical and non-surgical extractions as well as in single extractions (*p* <0.01) ( Table 2) as opposed to multiple extractions (*p* = 0.0693) ( Table 2). It is worth noting that most common post-operative signs and symptoms with moderate-severe intensity were not frequently reported at the 24-hour telephone appointment (dysphagia - 5.33 %, fever - 12.85%, swelling - 25.39%, trismus - 33.23%), whereas pain was perceived in approximately 73% of patients with various subjective intensities. This is probably related to the limited number of surgical extractions in this study which are those having the highest risk of complications, and to the use of antibiotic prophylaxis in a number of extractions.

According to the international literature (2-4,6,7,12), pain was the most common symptom reported: 72% of cases at 24 hours, and 51% at 72 hours, although almost always of a lower intensity, and in 18% at suture removal. In the control group, 33% of patients still complained of pain at suture removal with a mean intensity which was very similar to that of the test group (4.3 vs 4.1). In both groups, patients with inflammation, alveolar osteitis and infections at suture removal reported mild pain intensity (5) with the highest peak in alveolar osteitis, with a mean intensity of 7.5.

As many other authors have reported (2-4,6,7,13), dysphagia, trismus and swelling occurred post-operatively in almost all test group patients in the present study, with a peak within the first 24 hours. Afterwards, there was a decrease or stabilization up to 72 hours. After this time limit, almost all patients showed improvements, as long as, at the time of suture removal, more than 95% of patients in both groups did not complain about dysphagia, 82% did not complain about trismus and 78% did not complain about swelling.

At suture removal, in both groups symptoms decreased but did not disappear in all cases of infection and alveolar osteitis, whereas, in cases of inflammation, in the test group the percentage of patients with trismus decreased from about 90% at the first telephone appointment to about 46%, and the percentage of patients with dysphagia decreased from about 64% at the first telephone appointment to about 10%. In the control group, approximately 30% of patients with inflammation showed trismus at suture removal and only 6.57% showed dysphagia.

In patients with good healing and without complications, the percentage of those with trismus dropped from about 62% at the first telephone appointment to 11.61% at suture removal and the percentage of those with dysphagia dropped from 39.32% to 1.49%. In the control group, at suture removal, 2.67% of patients reported dysphagia and 14.28% reported trismus.

Approximately 80% of patients who had trismus, dysphagia and swelling at suture removal had third molars extracted, especially the lower ones; of these, 35% with trismus, 50% with dysphagia and 42% with swelling also did not have good healing.

The presence of symptoms should be accurately investigated at each telephone appointment since they were variously, but frequently, associated not only with simple alveolar inflammation but also with alveolar osteitis and infections.

Fever is not frequent in alveolar osteitis, however, temperatures above 38°C after the first 3 days have been associated with the presence of deep infections (14).

On the other hand, dysphagia and trismus are present in alveolar osteitis and especially in deep infections (2-4,6,7,15,16).

Swelling usually reaches its peak around day 3 and then regresses. (17) In case of its persistence and/or abnormal intensity, daily or even more frequent monitoring should be performed, thus hospitalization may be required (10,18,19), although hospitalization was not necessary for any patients in the present study.

Patient opinions on wound healing and whether or not they contact their family doctor for any problems are expression of the patient’s ability to self-diagnose themselves. Actually, the biggest problems in the telephone follow-up are represented by the patient’s inability to perform self-diagnosis and the interviewer’s difficulty to reach an evaluation of the actual surgical wound conditions through the patient’s description, as already reported for other kind of surgeries by Whitby *et al.* (20). Contrarily, Reilly *et al.* reported rather good patient capability of self-diagnosis (21), although they recommended examining the patient when he/she reports infection as well as when he/she is not sure of having infection.

In the present study, telephone follow-up was always carried out by the same surgeon (PA) so that, with her more in-depth history, she could identify the existence of problems which required the patient to come in for examination. Nevertheless, out of the 52 patients who, at the time of suture removal, had complications, only 39% said that the wound was not healing well at the 24-hour telephone appointment, indicating wound conditions consistent with those found at suture removal. Only 6% of patients without any complications at the time of suture removal thought that the wound was not healing well. All patients with alveolar osteitis and infections thought the wound was not healing well, while only 36% of patients with inflammation were able to detect a poor healing condition. All patients who were diagnosed with alveolar osteitis or infections at suture removal referred that the wound was not healing well at the 72-hour telephone appointment.

The percentage of patient self-diagnostic abilities in this study is similar to that reported in the literature (21), and it could likely be increased if patients were accurately informed about any possible signs or symptoms related to the normal post-operative progress before being discharged. If, on the one hand, this may worsen the patient’s self-diagnostic ability over time, on the other hand, it must be interpreted while taking into consideration that self-diagnosis of inflammation is more difficult to achieve than that of alveolar osteitis since symptoms are less pronounced, wound discomfort is often confused with that related to the presence of stitches, and since minor pain or discomfort are considered normal, especially in anxious patients who consider the extraction an invasive and complex procedure, even after being informed, thus expecting symptoms to be much worse. This problem is closely related to the possibility of increasing prevention through telephone follow-up: if the patient is the first to underestimate his/her own symptoms, it becomes difficult for the interviewer to intercept any complications and/or invite the patient to come in for a check-up before the suture removal appointment (20,21).

Phone contacts were made after 24 and 72 hours since post-operative symptoms usually increase during the first 48 hours and then start to progressively decrease (22).

The need for post-operative monitoring is supported by the fact that only 47% of patients who thought they were not healing well 24 and 72 hours after surgery felt they had to come in for examination before suture removal, thus making early diagnosis of alveolar osteitis and infection possible. This is probably due to the optimistic expectations of patients as far as spontaneous healing possibilities are concerned.

The discrepancy between the actual incidence of infections and the supposed need for a clinical exam makes an in-depth telephone inquiry fundamental in order to help the interviewer establish the real need to submit patients to a clinical examination.

According to previous studies (23,24), lower third molars were the teeth that were most frequently involved in inflammation, alveolar osteitis and infections (61% in the control group and 72% in the test group).

In the present study, the overall incidence of infections was 1.10% (0.63% for alveolar infections and 0.47% for alveolar osteitis), which was lower than that reported in other studies with or without antibiotic prophylaxis, which ranged from 1.2%-14.8% (25-27). However, alveolar inflammation occurred in 18.02% of cases but this complication has been rarely reported and discussed in the literature, probably due to its limited clinical significance (23,24).

In more than 70% of cases in which inflammation, alveolar osteitis and infections occurred, patients underwent antibiotic prophylaxis. Specifically, alveolar osteitis and infection occurred in 100% (7/7) of patients who underwent extraction of 1 lower third molar (with or without the ipsilateral upper molar during the same session) under antibiotic prophylaxis. Furthermore, more than 65% of cases (75/115) in which inflammation occurred were treated under antibiotic prophylaxis, about 75% of which (56) had a lower third molar extracted.

Although the antibiotic prophylaxis in the present study was not randomized but defined by each surgeon on the basis of personal clinical considerations since the study did not aim at verifying antibiotic effectiveness in avoiding surgical site infections, it may be still assumed, as opposed to the literature, that antibiotic prophylaxis was poorly effective. Actually, in about 54% of the sample that did not perform antibiotic prophylaxis, there was an 11% incidence of inflammation and no infections or osteitis occurred, although most extractions were non-surgical. This discrepancy may be related to the presence, in the cases in which antibiotics were administered, of local infectious risk conditions that motivated the need for antibiotic prophylaxis and which predisposed to infections anyway. However, antibiotic prophylaxis seems to not have been really effective in preventing alveolar osteitis and infections in lower third molar extractions, and this induces us to reflect on the appropriateness of prophylactic antibiotic administration in this procedure, contrary to what is affirmed by Cochrane’s most recent review on this issue (28), also considering the reported high number of patients needed to treat to avoid 1 case of infection, which exposes to the adverse effects of antibiotics and which increases the risk of bacterial resistance to antibiotics. It is likely that, in the present cases, contamination occurred after the antibiotic prophylaxis period and, therefore, it appears more logical to perform early diagnosis and therapy of an infection rather than carrying out unnecessary prophylaxis.

Telephone follow-up showed a high rate of satisfaction in patients (8.6 on a 0-10 scale) who, in more than 95% of cases, would have preferred not coming back in for follow-up if suture removal was not necessary, as opposed to the remaining patients who would have preferred coming back for clinical follow-up. Moreover, 56% of patients in the control group would have preferred being contacted by phone for follow-up. Patient satisfaction, also reported by Susarla *et al.* (11), and easy planning of phone calls which are not expensive for patients, interviewers, or for healthcare facilities, suggest that this kind of follow-up should always be carried out, both in public and in private healthcare facilities.

Findings of the present study support the theory that telephone follow-up seems to be a valid method of patient monitoring following tooth extractions, since it allows early detection of any possible wound healing complications, it is inexpensive, and it is widely well-accepted by patients.
